# Effect of Lipopolysaccharide-Induced Inflammatory Challenge on β-Glucuronidase Activity and the Concentration of Quercetin and Its Metabolites in the Choroid Plexus, Blood Plasma and Cerebrospinal Fluid

**DOI:** 10.3390/ijms22137122

**Published:** 2021-07-01

**Authors:** Małgorzata Domżalska, Wiesław Wiczkowski, Aleksandra Szczepkowska, Sylwia Chojnowska, Tomasz Misztal, Fruzsina R. Walter, Maria A. Deli, Hiroshi Ishikawa, Horst Schroten, Christian Schwerk, Janina Skipor

**Affiliations:** 1 Institute of Animal Reproduction and Food Research, Polish Academy of Sciences, 10-748 Olsztyn, Poland; m.domzalska@pan.olsztyn.pl (M.D.); w.wiczkowski@pan.olsztyn.pl (W.W.); a.szczepkowska@pan.olsztyn.pl (A.S.); 2 Faculty of Health Sciences, Lomza State University of Applied Sciences, 18-400 Lomza, Poland; schojnowska@pwsip.edu.pl; 3 The Kielanowski Institute of Animal Physiology and Nutrition, Polish Academy of Sciences, 05-110 Jabłonna, Poland; t.misztal@ifzz.pan.pl; 4 Institute of Biophysics, Biological Research Centre, ELKH, 6726 Szeged, Hungary; walter.fruzsina@brc.hu (F.R.W.); deli.maria@brc.hu (M.A.D.); 5 Laboratory of Regenerative Medicine, Department of Neurosurgery, University of Tsukuba, Tsukuba 305-8575, Ibaraki, Japan; ishi-hiro.crm@md.tsukuba.ac.jp; 6 Department of Pediatrics, Pediatric Infectious Diseases, Medical Faculty Mannheim, Heidelberg University, 68167 Mannheim, Germany; horst.schroten@umm.de (H.S.); christian.schwerk@medma.uni-heidelberg.de (C.S.)

**Keywords:** quercetin-3-glucuronide, quercetin, β-glucuronidase activity, choroid plexus, choroid plexus epithelial cells, cerebrospinal fluid, lipopolysaccharide, ewes

## Abstract

Quercetin-3-glucuronide (Q3GA), the main phase II metabolite of quercetin (Q) in human plasma, is considered to be a more stable form of Q for transport with the bloodstream to tissues, where it can be potentially deconjugated by β-glucuronidase (β-Gluc) to Q aglycone, which easily enters the brain. This study evaluates the effect of lipopolysaccharide (LPS)-induced acute inflammation on β-Gluc gene expression in the choroid plexus (ChP) and its activity in blood plasma, ChP and cerebrospinal fluid (CSF), and the concentration of Q and its phase II metabolites in blood plasma and CSF. Studies were performed on saline- and LPS-treated adult ewes (*n* = 40) receiving Q3GA intravenously (*n* = 16) and on primary rat ChP epithelial cells and human ChP epithelial papilloma cells. We observed that acute inflammation stimulated β-Gluc activity in the ChP and blood plasma, but not in ChP epithelial cells and CSF, and did not affect Q and its phase II metabolite concentrations in plasma and CSF, except Q3GA, for which the plasma concentration was higher 30 min after administration (*p* < 0.05) in LPS- compared to saline-treated ewes. The lack of Q3GA deconjugation in the ChP observed under physiological and acute inflammatory conditions, however, does not exclude its possible role in the course of neurodegenerative diseases.

## 1. Introduction

Quercetin (Q) is the most abundant dietary flavonoid, and its anti-oxidative and anti-inflammatory actions have been demonstrated in both in in vitro and in vivo conditions [1,2,3]. Of great interest is to utilize these beneficial properties of Q in the prevention and treatment of neurodegenerative disorders, such as Alzheimer’s and Parkinson’s disease, in which pathogenesis oxidative stress and inflammation play an important role [4]. To be potentially active in the brain, substances have to cross the blood–brain barrier (BBB) via the endothelium of brain microvessels and the blood–cerebrospinal fluid (CSF) barrier (BCSFB) via the epithelium of the choroid plexus (ChP) [5]. It was previously found in in vitro studies using BBB models that Q enters the brain [6,7]. The dietary Q occurs mainly as glycosides and, after hydrolysis to free Q aglycone in the gastro-intestinal tract, is rapidly metabolized via the phase II detoxification pathway (glucuronidation, methylation and sulphation), before reaching the target tissue, to less active or inactive metabolites, and in this form is also rapidly excreted via urine and bile [8]. Therefore, in human blood plasma, Q occurs mainly as Q-3-glucuronide (Q3GA), isorhamnetin-3-glucuronide (iRGA) and Q-3′-sulfate, while the Q aglycone is rarely detected [9,10]. The total concentration of Q (aglycone and metabolites) in human blood plasma is usually in the low nanomolar range, but after Q supplementation, can reach 1 µM [11], whereas the Q3GA concentration can reach about 260 nM [10]. Q3GA is considered to be a more stable form of Q for transport with the bloodstream to tissues, where it can potentially undergo an in situ deconjugation process and release any free Q aglycones [12,13]. The enzyme responsible for the catalytic detachment of the glucuronic acid residue form of β-D-glucuronides is β-glucuronidase (β-Gluc, EC 3.2.1.31), a lysosomal acid hydrolase [14]. Deglucuronidation often converts a pharmacologically inactive conjugate to an active aglycone; therefore, the biological effects of Q are proportional to its bioavailability and the rate of glucuronide deconjugation in tissues [15].

We demonstrated in an ovine model that the main metabolites of dietary Q in CSF are Q and its methylated form—izorhamnetin (iR) aglycones—whereas in the blood, Q3GA and iRGA dominate, but Q and iR aglycones are also present [16]. Two mechanisms explaining the Q aglycone presence in the CSF were considered: transport across the BCSFB via passive diffusion, and Q3GA deglucuronidation in the CSF. However, the last mechanism was questioned due to the lack of β-Gluc activity detected in the ovine CSF under experimental conditions [16]. Notably, the assumption that the ChP has potential for the deconjugation of Q3GA, resulted from: (1) the presence of β-Gluc in the ChP [17]; (2) distinct localization of Q3GA in the epithelial cells of the ChP suggesting its interaction with the BCSFB [18]; and (3) the presence of immune cells in the ChP stroma, mainly dendritic cells and macrophages [19]. As indicated in vitro, Q3GA binds to proteins on the surface of macrophages and is readily deconjugated to Q aglycone in basal conditions, which is further enhanced by lipopolysaccharide (LPS) treatment [20]. β-Gluc is released from lysosomes into the extracellular fluid, either as a result of cell damage or is secreted by neutrophils and macrophages at the site of ongoing inflammation [21,22]. Inflammatory activation of macrophages leads to an increase in β-Gluc activity due to enhanced lactate secretion and decreased extracellular pH (~4.5) associated with mitochondrial dysfunction [20]. Inflammation also up-regulates adhesion molecules and chemokines in the ChP and causes an increase in immune cells in the ChP stroma [23,24].

These initial findings allow us to hypothesize that inflammation on the periphery increases β-Gluc activity in the ChP and CSF and consequently leads to an increase in the concentration of Q in the CSF. Therefore, in the present study, we aimed to evaluate the effect of LPS-induced acute inflammation on: (1) β-Gluc gene expression in the ChP and its activity in blood plasma, the ChP and CSF; and (2) concentration of Q and its phase II metabolites in blood plasma and CSF. We also examined β-Gluc expression and activity, as well as Q3GA accumulation separately in the epithelial cells of the ChP under basal and LPS-challenged conditions.

## 2. Results

### 2.1. Effect of LPS-Induced Acute Inflammation on Gene Expression and Activity of β-Glucuronidase in the ChP (Exp. 1)

In in vivo studies, an increase in body temperature (Supplementary Figure S1A–C), plasma cortisol concentration (Supplementary Figure S1D–F) and sickness behavior manifested by dyspnea symptoms, sneezing, loose fecal consistency, lack of interest in food and reduced social interaction was observed in all LPS-treated ewes, which indicates a response to LPS from *Escherichia coli* 055:B5. The action of LPS on the ChP was further confirmed by the higher (*p* < 0.05) expression of C-C motif chemokine ligand 2 (*CCL2*) and C-X-C motif chemokine ligand 1 (*CXCL1*) in LPS- than in saline-treated ewes (Supplementary Figure S2).

**Figure 1:**
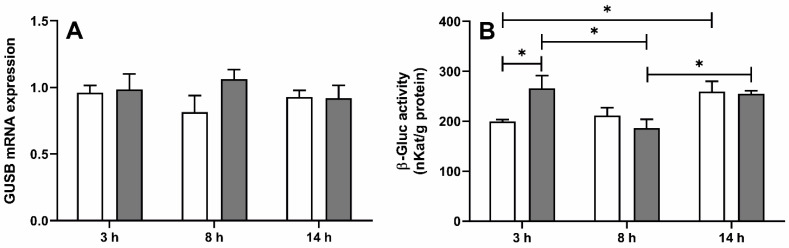
Mean (±SEM) relative mRNA expression of β-glucuronidase gene (*GUSB*) (**A**) and β-glucuronidase (β-Gluc) activity (**B**) in the choroid plexus in ewes euthanized 3 h (11:00 a.m., *n* = 8), 8 h (4:00 p.m., *n* = 8) and 14 h (10:00 p.m., *n* = 8) after saline (white bars) or lipopolysaccharide from *Escherichia coli* 055:B5 (grey bars) administration. * indicates significant differences at *p* < 0.05 (two-way ANOVA—Tukey’s post hoc test).

In the ovine ChP, the mean (± SEM) mRNA expression of *GUSB* (the gene encoding β-Gluc), determined by real-time PCR, was similar in both saline- and LPS-treated ewes (Figure 1A) at all investigated time points, whereas β-Gluc activity (Figure 1B) was (*p <* 0.05) higher in LPS-treated ewes but only 3 h after LPS administration (266.1 ± 50.4 vs. 199.8 ± 7.8 nKat/g of proteins). Moreover, in the saline-treated group, enzyme activity was significantly (*p* < 0.05) higher in ewes euthanized at 10 p.m. compared to those euthanized at 11 a.m.

### 2.2. Effect of LPS-Induced Acute Inflammation on β-Glucuronidase Activity and the Concentration of Q and Its Phase II Metabolites in Blood Plasma and Cerebrospinal Fluid (Exp. 2)

According to body temperature, blood plasma cortisol concentration (Supplementary Figure S3) and sickness behavior, all LPS-treated ewes responded to LPS. The function of BCSFB was not affected by LPS treatment, as indicated by a similar level of the mean Q-Alb quotient in saline- and LPS-treated ewes (Supplementary Figure S4).

**Figure 2:**
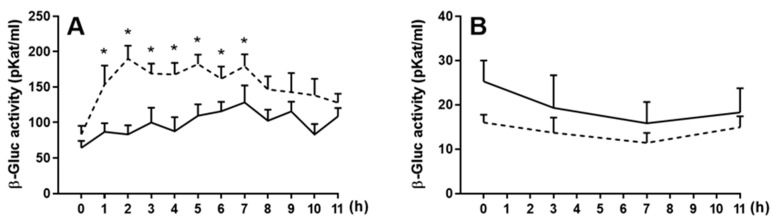
Mean (±SEM) β-glucuronidase (β-Gluc) activity in blood plasma (**A**) and cerebrospinal fluid (**B**) in ewes receiving saline (solid line) and then lipopolysaccharide (400 ng/kg of bw) (dotted line) after 2 weeks of recovery. * indicates significant differences at *p* < 0.05 (two-way ANOVA for repeated measures—Tukey’s post hoc test).

In the blood plasma of saline-treated sheep, β-Gluc activity did not change significantly through the entire period of the experiment, but in LPS-treated animals, the activity increased significantly (*p* *<* 0.05) from 73.8 ± 31.5 to 153.7 ± 75.3 pKat/mL in the first hour after LPS administration and stayed at a similar level through the next 6 h, and then decreased back to the level similar to that measured in saline-treated ewes (Figure 2A). In CSF, there was no difference between β-Gluc activity in saline- and LPS-treated ewes (19.7 ± 16.7 vs. 14.1 ± 7.5 pKat/mL, respectively) through the entire period of the experiment (Figure 2B).

**Figure 3:**
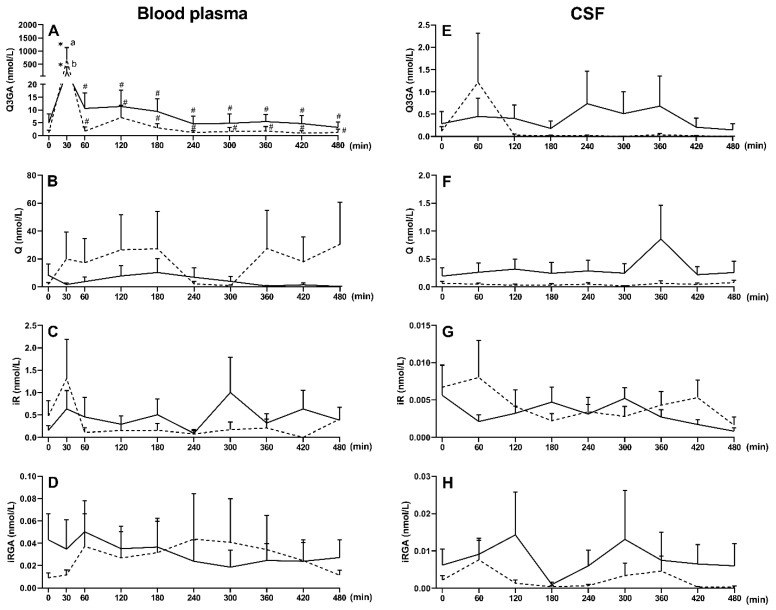
Mean (±SEM) concentration of Q3GA (**A**,**E**), Q aglycone (**B**,**F**), iR (**C**,**G**) and iRGA (**D**,**H**) in blood plasma and CSF of saline-treated (*n* = 10, solid line) and LPS-treated (*n* = 10, dotted line) ewes after an intravenous administration of Q3GA at dose of 6 mg/kg bw. Different lowercase letters indicate significant differences between saline- and LPS-treated ewes (two-way ANOVA for repeated measures—Sidak post hoc test), * indicates significant differences compared to time 0 (two-way ANOVA for repeated measures—Dunnett post hoc test), # indicates significant differences compared to 30 min (two-way ANOVA for repeated measures—Dunnett post hoc test), *p* < 0.05.

Before Q3GA administration, the presence of Q (8.4 ± 8 and 1.8 ± 1.3 nmol/L), and iR (0.2 ± 0.1 and 0.5 ± 0.3 nmol/L) aglycones and their monoglucuronides, Q3GA (4.9 ± 3.6 and 1.1 ± 1 nmol/L) and iRGA (0.04 ± 0.02 and 0.009 ± 0.004 nmol/L), was observed in saline- and LPS-treated ewes, respectively (Figure 3). Intravenous administration of Q3GA at a dose of 6 mg/kg body weight (bw) resulted in a significant (*p* < 0.05) increase in Q3GA concentration in plasma in saline- and LPS-treated ewes (Figure 3A), with concentrations achieved 30 min after Q3GA administration being higher in LPS- than saline-treated ewes (675 ± 458 nmol/L and 210 ± 180 nmol/L, respectively). Subsequently, as a result of extensive metabolism and elimination, there was a sharp decrease in Q3GA concentration. There were, however, no significant changes in Q, iR, and iRGA concentrations compared to the period before Q3GA administration (Figure 3B–D). No sulphated derivatives (Q-sulf, iR-sulf, Q-gluc-sulf and iR-gluc-sulf) were found in the blood plasma before or after Q3GA administration. Q3GA dominated in the blood plasma, reaching 84.4% of the total concentration of Q in the saline-treated and 79.8% in the LPS-treated ewes, while the participation of Q aglycone was 14.15% and 19.79%, iR 1.40% and 0.37% and iRGA 0.1% and 0.03%, respectively (Supplementary Table S1).

In the CSF collected before Q3GA administration, Q, iR, Q3GA and iRGA, but no sulphated derivatives, were found in saline- and LPS-treated ewes. After Q3GA administration, no significant change in their concentration was observed in ewes treated with saline or LPS (Figure 3E–H). Additionally, the percentages of Q and its metabolites in relation to the total Q content remained similar before and after Q3GA administration. The CSF was dominated by Q3GA (54.7 vs. 74.8%), while the participation of Q was 43.8% and 22.2%, iR 0.4% and 1.94% and iRGA 1.06% and 1.04%, respectively, in saline and LPS-treated ewes (Supplementary Table S2).

**Figure 4:**
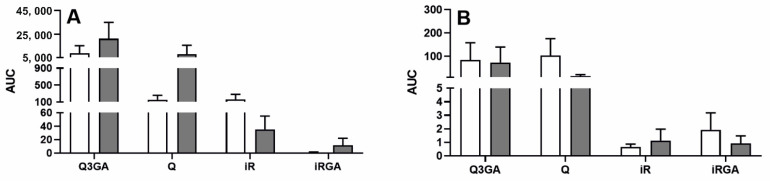
Mean (±SEM) area under the curve (AUC) of Q3GA, Q, iR and iRGA concentration over time in the blood plasma (**A**) and cerebrospinal fluid (**B**) of saline-treated (*n* = 10, white bars) and LPS-treated (*n* = 10, grey bars) ewes after an intravenous administration of Q3GA at dose of 6 mg/kg bw.

In both blood plasma (Figure 4A) and CSF (Figure 4B), there was no effect of acute inflammation caused by intravenous LPS administration on the total content of Q3GA, Q, iR and iRGA, measured by the area under the concentration curve (AUC) of Q3GA, Q, iR and iRGA over time. The mean AUC of the concentrations of Q3GA, Q, iR and iRGA in the CSF was approximately 70- and 180-fold lower than the AUC of blood plasma concentrations of these compounds in the saline- and LPS-treated ewes, respectively.

### 2.3. Effect of LPS on Gene Expression and Activity of β-Glucuronidase (Exp. 3) and Q3GA Accumulation in the Epithelial Cells of the Choroid Plexus In Vitro (Exp. 4)

**Figure 5:**
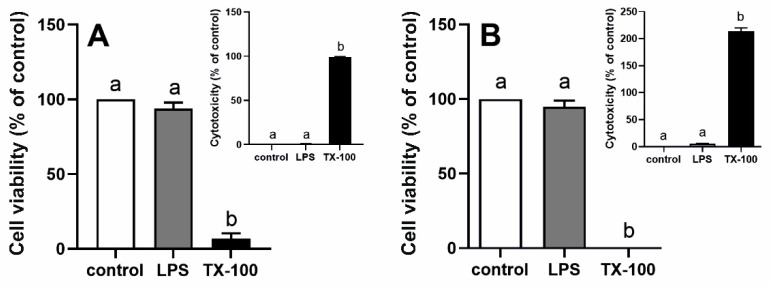
Mean (±SEM) rat choroid plexus epithelial cells (**A**) and HIBCPP cells (**B**) viability and lipopolysaccharide (LPS) cytotoxicity (inserted) after 48 h incubation with saline (control, white bars), LPS (grey bars) or Triton X-100 (TX-100, black bars), (*n* = 4). The absorbance results are presented as percentage values (%), where the control group (cell viability) and TX-100 (cytotoxicity) are considered as 100%. Different lowercase letters indicate significant differences at *p* < 0.05 (one-way ANOVA).

In in vitro studies with epithelial cells of rat ChP and human ChP papilloma (HIBCPP) cells, the action of LPS (5 µg/mL) from *Escherichia coli* O111:B4 was confirmed by higher (*p* < 0.05) CXCL1 and interleukin 6 (*IL6)* gene expression in LPS-treated rat epithelial cells (Supplementary Figure S5B,C) or by higher (*p* < 0.05) interleukin 8 (*IL8*) gene expression in LPS-treated HIBCPP cells (Supplementary Figure S6B) than in their saline-treated controls. LPS at a dose of 5 µg/mL did not affect these cells’ viability and was not toxic, as indicated on Figure 5A,B and inserts, respectively.

**Figure 6:**
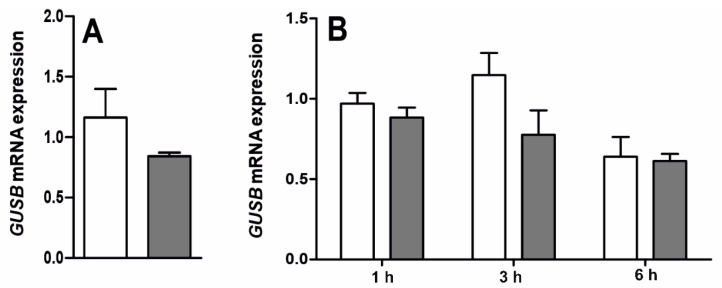
Mean (±SEM) relative mRNA expression of β-glucuronidase (*GUSB*) gene in rat choroid plexus epithelial cells (**A**) incubated for 3 h with saline (white bars) or lipopolysaccharide (LPS, grey bars) and in HIBCPP cells (**B**) incubated for 1, 3 and 6 h with saline/LPS, (*n* = 3).

**Figure 7:**
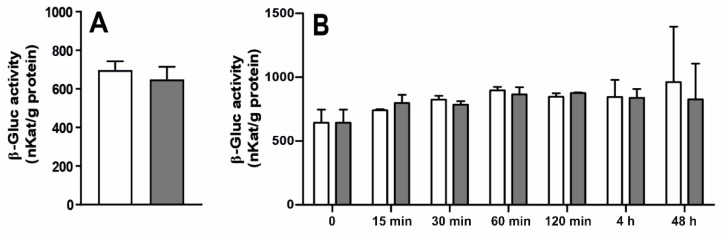
Mean (±SEM) β-glucuronidase (β-Gluc) activity in rat choroid plexus epithelial cells (**A**) incubated for 3 h with saline (white bars) or lipopolysaccharide (LPS, grey bars) and in HIBCPP cells (**B**) incubated for 15; 30; 60 and 120 min and 4 and 48 h with saline/LPS, (*n* = 3).

In rat ChP epithelial cells, as well as HIBCPP cells, the mean (± SEM) mRNA expression of *GUSB* (Figure 6A,B) and β-Gluc activity (Figure 7A,B) was similar in both saline- and LPS-treated cells at all investigated time points. Moreover, there was no β-Gluc activity in the medium collected from the culture of epithelial cells of rat ChP (after 3 and 6 h) and HIBCPP cells (after 3, 6 and 24 h), either in the saline- or LPS-treated cells.

**Figure 8:**
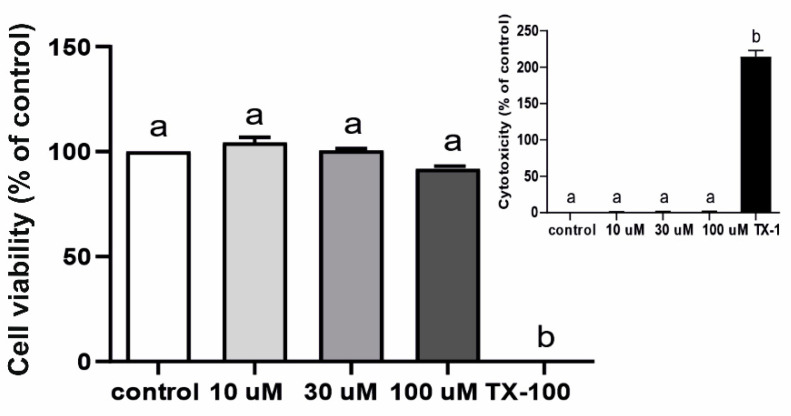
Mean (±SEM) HIBCPP cell viability and Q3GA cytotoxicity (insert) after 48 h incubation with saline (white bars), 10 µM, 30 µM and 100 µM Q3GA (grey bars) or Triton X-100 (TX-100, black bars) (*n* = 3). The absorbance results are presented as percentage values (%), where the control group (cell viability) and TX-100 (cytotoxicity) are considered as 100%. Different lowercase letters indicate significant differences at *p* < 0.05 (one-way ANOVA).

As indicated in Figure 8, Q3GA in the doses of 10 µM, 30 µM and 100 µM did not affect HIBCPP cell viability and was not toxic.

**Figure 9:**
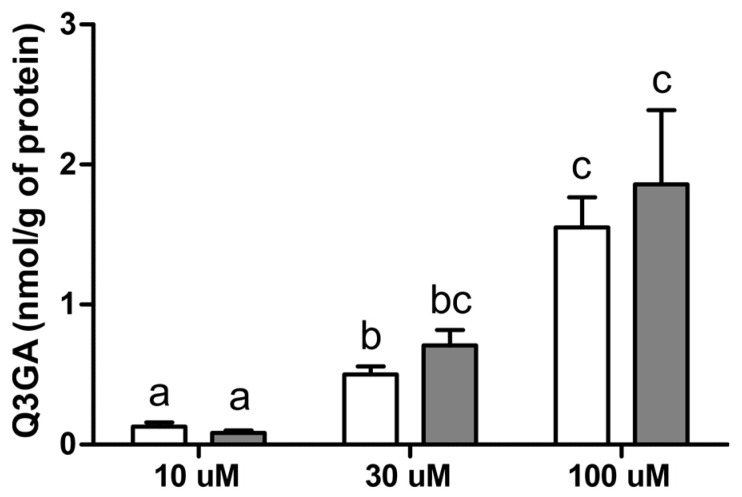
Mean (±SEM) Q3GA concentration in saline- (white bars) and LPS-treated (grey bars) HIBCPP cells incubated with 10, 30 and 100 µM Q3GA for 30 min, (*n* = 5). Different lowercase letters indicate significant differences at *p* < 0.05 (two-way ANOVA—Tukey’s post hoc test).

We observed that in HIBCPP cells incubated with 30 µM of Q3GA, the concentration of Q3GA was significantly (*p* < 0.05) higher when cells were harvested mechanically (307.1 ± 43.7 ng/mL) compared to enzymatically (20.3 ± 5.1 ng/mL). In HIBCPP cells, after 30 min incubation with an increasing dose of Q3GA (10, 30 and 100 µM), a significant (*p* < 0.05) increase in Q3GA concentration was observed in both saline- and LPS-treated cells (0.13 ± 0.03; 0.5 ± 0.06 and 1.55 ± 0.22 vs. 0.08 ± 0.02; 0.71 ± 0.11 and 1.86 ± 0.53 µmol/g protein, respectively), which was positively correlated with the dose of Q3GA (r = 0.9449 for the saline- and r = 0.8315 for the LPS-treated group). No effect of LPS was observed for Q3GA accumulation in either the saline- or LPS-treated HIBCPP cells (Figure 9).

## 3. Discussion

This is the first study to comprehensively evaluate the effect of LPS-induced acute inflammation on β-Gluc activity in the ChP and the CSF, together with the concentration of Q and its phase II metabolites in the CSF. Studies were carried out on a sheep model previously used to evaluate flavonoid access to the central nervous system (CNS) [16,25,26,27] and the ChP response to LPS-induced acute inflammation [28,29,30,31]. We have provided evidence that inflammation caused by intravenous LPS administration influences β-Gluc activity in the ovine ChP but does not affect *GUSB* mRNA expression. Considering epithelial, endothelial and stroma resident immune cells that compose the ChP, macrophages seem to be responsible for the observed increase in β-Gluc activity in the ChP. According to Ishisaka et al. [20], inflammatory stimuli increase β-Gluc activity (measured by deconjugation of Q3GA), but not β-Gluc gene expression and protein secretion in primary and cell line macrophages, and does not affect the enzyme activity in endothelial cell lines, such as human umbilical cord vein endothelial cells (HUVEC) and bovine aorta endothelial cells (BAEC). Our results indicate that LPS also does not affect either *GUSB* or β-Gluc activity in epithelial cells, as indicated in primary cultures of rat ChP and human ChP epithelial papilloma cells (HIBCPP). Interestingly, the increase in β-Gluc activity in the ChP was observed 3 h after LPS administration, but not later (8 and 14 h). This early increase in β-Gluc activity does not appear to be connected with the infiltration of immune cells into the ChP. In the ovine ChP, we observed strong increase in *CCL2* and *CXCL1* expression 3 h after LPS administration, and a similar increase in CCL2 in sheep could be suggested due to the correlation between *CCL2* mRNA expression and CCL2 protein levels in the CSF previously demonstrated in rats [32]. Therefore, immune cells can be expected to infiltrate the ChP thereafter, which means that an increase in β-Gluc activity could also be expected later than 3 h after LPS administration. There is also the possibility that a temporary increase in β-Gluc activity in the ChP may result from the presence of blood in the vascular bed. The ChP is a richly vascularized structure with high blood flow [33], and the results of experiment 2 showed that the activity of β-Gluc in blood plasma was higher in LPS- than in saline-treated ewes, particularly in the period between 2 and 7 h after LPS administration. These results are consistent with observations indicating that the β-Gluc activity is greater in the blood plasma of LPS-stimulated rats and mice [22,34,35]. There is also a possibility that the LPS-induced activation of ChP residing macrophages triggered lactate secretion and induced local acidification, as was observed during LPS stimulation of the RAW264 cells [20]. This, in turn, may increase β-gluc activity in the ChP, because β-gluc as lysosomal enzyme requires the acidic conditions for its catalytic activity [14].

We also detected β-Gluc activity in the CSF in both saline- and LPS-treated ewes, while our previous studies [16] indicated no β-Gluc activity in the ovine CSF under physiological conditions, which may be linked with the methods used in both studies. The low activity of β-Gluc in the CSF in ewes is consistent with the reports of Beratis et al. [36] describing the presence of lysosomal exoglycosidases, including β-Gluc, in CSF in humans. In contrast to blood plasma, the activity of β-Gluc in the ovine CSF did not differ between saline- and LPS-treated ewes through the entire period of experiment 2. The lack of correlation between β-Gluc activity in the blood plasma and in the CSF indicates that this enzyme did not enter the CSF from blood. Most likely, it is released/secreted from cells present in the CSF, including cells of the immune system. According to Long et al. [37], there is a high degree of correlation between the activity of β-Gluc (as an indicator of inflammation) and the total concentration of protein, albumin and globulin in CSF (as an indicator of changes in BCSFB permeability). We did not observe differences between β-Gluc activity and the albumin quotient (Q-Alb) between saline- and LPS-treated ewes, which indicates the lack of inflammation in the CNS as well as no changes in BCSFB permeability during the entire period of experiment.

In the present study, the accumulation of Q3GA in the human ChP epithelium previously demonstrated with the use of immunohistochemistry [18] was further confirmed in HIBCPP cells by the HPLC-MS-MS method. The observed significantly higher concentration of Q3GA in the homogenate of HIBCPP cells obtained by the mechanical vs. enzymatic method proves that Q3GA binds on the surface of cells, but does not penetrate into the cell. The amount of Q3GA accumulated on the HIBCPP cells surface after 30 min incubation with 10 µM Q3GA was in the same range as that detected in macrophage RAW264 cells (60–75 pmol/mg of proteins), but higher than in rat brain endothelial cells (RBEC1 line, 6 pmol/mg of proteins) at 30 min of incubation with 20 µM Q3GA [18]. In contrast to macrophages, where the accumulation of Q3GA was increased following LPS stimulation [10], Q3GA accumulation on HIBCPP cells was not affected by LPS. Moreover, we observed no β-Gluc activity in the medium obtained from HIBCPP cell culture in basal and LPS-stimulated condition, which indicates that these cells do not secrete β-Gluc and/or were not damaged by LPS (confirmed by toxicity tests). This shows that it is the macrophages rather than choroidal epithelial cells which play a key role in the binding of Q3GA and serve as a potential Q3GA pool and its deconjugation site during inflammation. The model used in the present study enabled evaluation of the effect of acute inflammation on the concentration of Q and its metabolites simultaneously in the blood plasma and in the CSF in the same animals injected intravenously (iv) with Q3GA. The ewes were first treated as a control group and received Q3GA; after several days, the same ewes were treated with LPS to induce inflammation, and after its development, they received Q3GA. Small amounts of Q3GA, Q aglycone, as well as trace amounts of iR and iRGA, but no sulphated derivatives, were present in the blood plasma and the CSF prior to Q3GA injection in control and LPS-treated ewes. Our previous studies [16] indicated sulphated Q conjugates as well as Q and iR aglycone in the blood plasma in onion-fed ewes, but in an amount much smaller compared to glucuronidated derivatives. In the present experiment, 24 h fasting resulted in a decrease in sulphated metabolites to values below the sensitivity of the method used, but did not fully eliminate Q and other metabolites. Similar difficulties with the elimination of Q were observed in rats, where despite the use of a special diet with a very reduced content of Q, trace amounts of this flavonoid were present in the brain [38]. As was expected, the blood plasma concentration of Q3GA increased following its intravenous administration. Interestingly, the concentration of Q3GA measured 30 min after its iv injection in ewes that developed acute inflammation was significantly higher compared to that observed in control ewes (675 ± 458 vs. 210 ± 180 nmol/L, respectively). A similar phenomenon occurred in another experiment performed by us (data not shown here). This may indicate an effect of acute inflammation on the volume of distribution and/or clearance of Q3GA; however, further studies are necessary to explain this. In the present study, the percentage of Q and iR aglycones as well as their respective monoglucuronides, Q3GA and iRGA, in the CSF differed compared to that found in the CSF in ewes receiving the dry onion skin suspension intraruminally [16]. In the CSF collected after Q3GA administration, Q3GA and Q aglycone dominated in both physiological and inflammatory conditions (54.7 and 74.8% for Q3GA and 43.8 and 22.2% for Q, respectively), and iR and iRGA were less abundant, whereas in ewes fed with dry onion skin, Q (55%) and iR (33%) aglycones dominated, and their monoglucuronides accounted for 12% of the total Q concentration. In the present study, an increased plasma concentration of Q3GA did not result in an increase in Q3GA in the CSF, which indicates that in ewes this metabolite is unlikely to pass through the BCSFB, and the presence of Q3GA in the CSF may be due to the transport of Q3GA as a product of Q glucuronidation in the cell. Increased plasma concentration of Q3GA did not result in the increase in Q aglycone under basal and inflammatory conditions (expected after the elevation of β-Gluc activity during LPS-induced acute inflammation), which could explain the lack of increase in the concentration of Q and IR in the CSF. These data taken together do not support the deconjugation of Q3GA in the ChP, either under basal or acute inflammation conditions. It should be emphasized, however, that this does not exclude the possibility of local Q3GA deglucuronidation in the course of neurodegenerative diseases characterized by chronic inflammation and having mitochondrial dysfunction in their etiology. This issue requires further studies.

## 4. Materials and Methods

### 4.1. Animals and Experimental Design

Studies were performed on adult female cross breed sheep (*n* = 40, 3–5 years old, 50–70 kg body weight (bw), body condition score 3 (rated on a scale of 1 to 5) maintained indoors and fed a constant diet of commercial concentrates with hay and water available ad libitum. Experimental protocols were approved by the Local Ethics Committee in Olsztyn (14/2015, Exp. 1) and the II Local Ethics Committee in Warsaw (WAW2/48/2016, Exp. 3 and Exp. 5) and performed in accordance with the Polish Guide for the Care and Use of Animals. In accordance with the European Union Directive 2010/63/EU, male Wistar rats (*n* = 24, 8 weeks old) were sacrificed with the use of xylazine/ketamine for tissue ChP collection for further epithelial cells isolation.

Experiment 1 was designed to evaluate the effect of LPS-induced acute systemic inflammation on the mRNA expression of *GUSB* and β-Gluc activity in the ChP. Studies were performed on 24 ewes. On the morning of the experiment, a catheter was inserted into the jugular vein to allow for blood sampling and substance administration without disturbing the animals. Afterwards, the sheep were placed in individual cages where they could lie down and have access to water and visual contact with each other in order to avoid isolation stress. The sheep were randomly divided into two groups and were treated intravenously (iv) with 0.9% *w*/*v* NaCl (saline-treated, *n* = 12) and with LPS (LPS-treated, *n* = 12) from *Escherichia coli* 055:B5 (Sigma-Aldrich, St. Louis, MO, USA) at a dose of 400 ng/kg of bw (dissolved in saline at a concentration of 10 mg/L (10 µg/mL)), as has been used previously to induce acute systemic inflammation in ewes [29]. The body temperature was measured, and blood was sampled 1 h before and every hour after NaCl/LPS administration. After centrifugation in heparinized tubes, blood plasma was stored at −40 °C until assay for cortisol concentration necessary for the validation of animal responses to LPS-induced inflammation. The sheep (twelve from the saline-treated and twelve from the LPS-treated group) were euthanized 3 h (11:00 a.m., *n* = 8), 8 h (4:00 p.m., *n* = 8) and 14 h (10:00 p.m., *n* = 8) after NaCl/LPS administration. After sacrifice, they were decapitated, and the ChP from lateral and third ventricles was isolated and then immediately frozen in liquid nitrogen and stored at −80 °C until further analyses of *GUSB* mRNA expression and β-Gluc activity as well as the mRNA expression of chemokines for the validation of ChP responses to LPS-induced inflammation.

**Figure 10:**
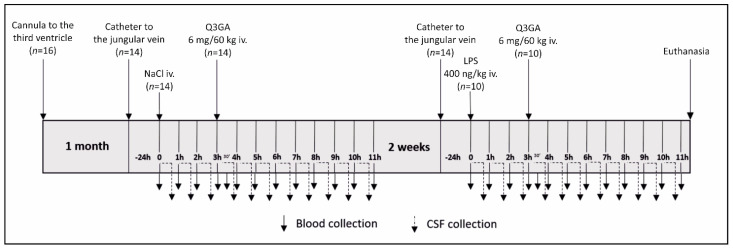
Schematic diagram of experiment 2. One month after guide cannula implantation to the third ventricle of the brain, a catheter to the jugular vein was inserted, and the next day, after the first samples of blood and cerebrospinal fluid (CSF) were collected, the sheep were treated intravenously (iv) with saline and after next 3 h with quercetin-3-glucuronide (Q3GA) at a dose of 6 mg/60 kg of body weight. After 2 weeks of recovery, the sheep were treated as before but instead of saline, they received iv lipopolysaccharide (LPS) at a dose of 400 ng/kg of body weight. Blood (solid arrows) and CSF samples (dotted arrows) were continuously collected every 1 h for 11 h after saline/LPS administration (blood was additionally sampled 30 min after Q3GA administration). After the end of experiment, the sheep were euthanized.

Experiment 2 was designed to evaluate the effect of LPS-induced inflammation on β-Gluc activity and the concentration of Q and its phase II metabolites in blood plasma and CSF. Studies were performed on sheep (*n* = 16) according to a repeated measures design, where the subjects were their own controls. However, 6 ewes were excluded from sampling: 2 ewes from the first session due to the health problems after guide cannula implantation, and 4 ewes from the second session due to problems with CSF collection. A schematic diagram of the experiment is presented in Figure 10. One month before the experiment, ewes were implanted with stainless-steel guide cannulas in the third ventricle of the brain for the long-lasting collection of CSF, as described earlier [39]. On the morning of the experiment, ewes were implanted with the jugular vein catheter and placed in individual cages. To collect samples of CSF, the stainless-steel catheter was introduced into the guide cannula and connected to a special cannula–Eppendorf tube system joined to the CMA 402 Syringe Pump (CMA, Stockholm, Sweden). After the first samples of blood and CSF were collected, the ewes were iv-treated with saline and after the next 3 h with Q3GA (Sigma Aldrich, St. Louis, MO, USA) at a dose of 6 mg/60 kg of bw (corresponding to ~4 mg of Q aglycone), dissolved in 50 °C saline and further samples of blood and CSF were collected for 8 h. After the end of the first part of experiment, the jugular vein and stainless-steel catheters were removed, and the ewes recovered for 2 weeks. In the second part of experiment, the sheep were treated as in the first part but with one exception. Instead of saline, they received iv LPS (at a dose of 400 ng/kg of bw). The body temperature was measured every hour, starting 1 h before saline/LPS administration. Blood was sampled every hour for a period of 11 h and additionally 30 min after Q3GA administration and then centrifuged in heparinized tubes and stored at −40 °C for further analysis. CSF from the third ventricle was collected continuously for 11 h at a rate of 20 mL/min, and immediately after filling, the tubes were stored at −40 °C until further analysis. Samples from ewes participating in both sessions qualified for further analysis After the end of experiment, the sheep were euthanized.

Experiment 3 was designed to evaluate the effect of LPS-induced inflammation on *GUSB* expression and β-Gluc activity in the epithelial cells of the ChP. The following experiment was carried out on the rat ChP epithelial cells and human ChP papilloma (HIBCPP) cells established by Ishiwata et al. [40]. Rat ChP epithelial cells were isolated in accordance with the procedure described by Monnot and Zheng [41]. Briefly, isolated ChPs were pulled and then minced and digested by 0.2% pronase (Sigma-Aldrich, St. Louis, MO, USA) to obtain ChP epithelial cells which were seeded on collagen-coated 6-well plates (n = 3 for each treatment) (TPP, Bionovo, Trasadingen, Switzerland) (0.5–1 × 10^6^ cells/well) and cultivated in DMEM/F12 with 2.5 mM L-glutamine (Gibco 31330-095, Thermo Scientific, Waltham, MA, USA), supplemented with 10% heat-inactivated fetal bovine serum (FBS, Thermo Scientific, USA), 5 µg/mL ITS (PAN Biotech, Poland), penicillin (100 U/mL)/streptomycin (100 µg (Sigma-Aldrich, St. Louis, MO, USA), 2.5 µg/mL amphotericin B (Sigma-Aldrich, St. Louis, MO, USA) and 10 ng EGF (Sigma-Aldrich, St. Louis, MO, USA). Transferring it to a medium with epithelial cells after the fibroblasts had been attached (about 8 h) and the addition of cis-hydroxy-D-proline (Sigma-Aldrich, St. Louis, MO, USA) eliminated fibroblasts from the cell culture. The purity of the cell population and the correct collection of the ChP was verified by analyzing the expression of *TTR* mRNA, the high level of which is characteristic for the ChP and its epithelial cells (Supplementary Figure S5A). HIBCPP cells were seeded on 6-well plates (n = 3 for each treatment and time point) and cultivated until confluence in DMEM/F12 with L-glutamine, supplemented with 10% FBS, 5 µg/mL ITS, penicillin (100 U/streptomycin (100 µg/mL) and 2.5 µg amphotericin B. For experiments, the medium was removed, and cells were washed with PBS and NaCl or LPS (*Escherichia coli* O111:B4, Sigma-Aldrich, St. Louis, MO, USA) were added to the fresh medium, at a dose of 5 µg/mL, which has been indicated to affect cells in vitro (Supplementary Figure S6B). We have previously shown that LPS does not activate nuclear factor kappa B inhibitor zeta *(NFKBIZ)*, *IL6* or *IL8* in HIBCPP cells [42]. The variation in cellular response could be due to the source of LPS used (compare with Supplementary Figure S6A provided by Julia Borkowski) or differences during the culture of the HIBCPP cells. Rat ChP epithelial cells were harvested mechanically after 3 h and HIBCPP cells after 15 min; 30 min; 1 h, 2 h, 3 h, 4 h, 6 h and 48 h after NaCl/LPS administration to determine the expression of *GUSB* and *CXCL1*, *IL6* and *IL8*, enabling the assessment of cell responses to LPS. To measure β-Gluc activity, cells were cultured in DMEM/F12 medium without phenol red (Gibco 11039-047, Thermo Scientific, Waltham, MA, USA) and the cells and medium were collected as described before.

Experiment 4 was designed to evaluate the effect of LPS-induced inflammation on Q3GA accumulation in the ChP epithelial cells. A preliminary experiment was performed to determine the effect of cell isolation on Q3GA accumulation. HIBCPP cells were seeded on 6-well plates (*n* = 3 for each method), and after reaching 80% confluence, the medium was changed with DMEM/F12 containing 30 μM Q3GA (Stock I (100mM Q3GA) in methanol was used to prepare Stock II (1mM Q3GA) by diluting stock I in medium and the final solution (30 µM Q3GA) was prepared by adding of stock II to the medium). After 30 min incubation, cells were separated from the plate enzymatically (0.25% trypsin) or mechanically (scraping in PBS) (38). Harvested cells were then frozen and stored at −80 °C until further HPLC/MS/MS analysis. For Q3GA accumulation studies, HIBCPP cells were seeded on filter inserts (*n* = 5 for each treatment) (ThinCert™ Cell Culture Inserts 6 Well, pore diameter 0.4 µm, pore density 1 × 10^8^ pores per cm^2^, growth area 4.5 cm^2^, translucent, Greiner Bio-One GmbH, Frickenhausen, Germany)—inverted culture and cultivated until confluence in DMEM/F12 with L-glutamine with 10% FBS and ITS, penicillin/streptomycin and amphotericin B, as described in Experiment 3. After reaching a confluence of 90%, confirmed by an increase in transepithelial electrical resistance (TEER), the medium was changed to phenol red free medium (Gibco 11039-047, Thermo Scientific, Waltham, MA, USA) without FBS. In the experimental group, LPS (*Escherichia coli* O111: B4) was added to the medium inside the insert at a dose of 5 µg/mL and in the control group 0, 9% NaCl. After 3 h of incubation, the medium in the insert was changed with DMEM/F12 containing 10 μM, 30 μM and 100 μM Q3GA. Thirty minutes after the addition of Q3GA, the medium was removed by pipetting and cells were washed three times with PBS and then separated from the insert membrane by a mechanical method (scraping in PBS with a small cell scraper), gently collected by pipetting, pelleted by centrifugation, and then frozen and stored at −80 °C until further HPLC/MS/MS analysis.

### 4.2. Analytical Methods

#### 4.2.1. Cortisol, Protein Level and β-Glucuronidase Activity

To validate animal responses to LPS treatment in both experiments, the concentration of cortisol in blood plasma was assayed by RIA using a method described by Kokot [43]. The sensitivity of the assay for cortisol was 0.95 ng/mL, and the intra- and inter-assay coefficients of variation were 10% and 12%, respectively.

Total protein concentrations in tissue homogenates and cell lysates were determined using the BCA Protein Assay Kit (BCA, Sigma-Aldrich, St. Louis, MO, USA). The assay was conducted according to the manufacturer’s protocol.

The activity of β-Gluc in the blood plasma, CSF, ChP and ChP epithelial cells was measured by the rate of p-nitrophenol released from p-nitrophenyl-β-d-glucuronide (Sigma-Aldrich, St. Louis, MO, USA) according to the method described by Chojnowska et al. [44]. The samples of ChP were rinsed in 0.9% saline (3 times) and then weighed, suspended in 0.15 M KCl with 0.2% Triton X-100 (9 mL of fluid for 1 g of tissue), homogenized using a FastPrep24 instrument (MP Biomedicals, Illkirch-Graffenstaden, France), and inserted into Lysing Matrix D tubes (MP Biomedicals, Illkirch-Graffenstaden, France). Homogenates were centrifuged (12,000× *g* rpm, 20 min, 4 °C) and the supernatants were used to determine enzyme activity. Due to the presence of hemoglobin in tissue, the method of β-Gluc measurement was modified and the method using the trichloroacetic acid (TCA) described by Chojnowska et al. [45] was used. The reaction mixture containing 50 μL of supernatant from homogenized tissues, 150 μL of substrate and 200 μL of acetate buffer (pH 4.5) was incubated for 1 h at 37 °C with continuous shaking. After incubation, 5 μL of a saturated solution of TCA was added to the mixture to precipitate the proteins. After centrifugation (14,000× *g* rpm, 5 min, RT), supernatants (80 μL) were collected, plated on a 96-well plate, and 200 μL of borate buffer (pH 9.8) was added to stop the enzymatic reaction. To measure the β-Gluc activity in blood plasma/CSF/cells, 30 µl substrate solution (6.3 mM p-nitrophenyl-β-d-glucuronide) in 40 µL 100 mM acetate buffer (pH 4,5) was added to 10 µL of blood plasma/CSF/cell-free extract. Incubation was carried out in 96-well microplates at 37 °C for 1 h with constant shaking and the reaction was terminated by 200 μL of 200 mM borate buffer (pH 9.8). After incubation of the reaction mixtures, the released 4-nitrophenol was measured at 410 nm (Epoch Microplate Spectrophotometer, Biotek Instruments, VT, USA) against the reaction mixture without tissue/cell supernatant/blood plasma/CSF, and β-Gluc activity was expressed in nKat/g protein or pKat/mL. The p-nitrophenol concentration was determined from a standard curve of 0.3 mM p-nitrophenol (Sigma-Aldrich, St. Louis, MO, USA) in 200 mM borate buffer (pH 9.8). The sensitivity of the assay for β-Gluc activity was 0.003 pKat/mlL.

#### 4.2.2. Cell Viability Assay (MTT) and Cytotoxicity Assay (LDH)

The MTT and LDH assays were performed to determine the viability and cell membrane integrity of rat ChP epithelial cells and HIBCPP cells under experimental conditions. Assays were performed using the Vybrant MTT Cell Proliferation Assay Kit (Thermo Scientific, Waltham, MA, USA) and the Pierce LDH Cytotoxicity Assay Kit (Thermo Scientific, Waltham, MA, USA). The assays were conducted according to the manufacturer’s protocol. The cells were seeded in 96-well plates (TPP, Bionovo, Trasadingen, Switzerland) until the cell monolayer become confluent. The medium was removed, and cells were washed with PBS and exposed to LPS (5 µg/mL) or Q3GA (10 µM, 30 µM or 100 µM). Cells were incubated for 48 h at 37 °C. Control groups were processed equally and incubated without LPS or Q3GA simultaneously. Cell viability was expressed as a percentage of the viability of control cells, assigned as 100%. To determine total LDH activity, cells from the positive control group were treated with 1% Triton X-100. After treatment, samples were analyzed using a commercial LDH kit following the manufacturer’s recommendations, and LDH activity was expressed as a percentage of the control, which was Triton X-100 treatment.

#### 4.2.3. Western Blotting and CSF/Blood Plasma Quotient

To assess the functions of BCSFB albumin quotient (Q-Alb) was used. The integrity of BCSFB was estimated by the ratio of albumin concentrations in CSF and blood plasma using a method described earlier by Skipor et al. [28]. The albumin quotient was evaluated as follows: Q − Alb = Alb (CSF)/Alb (blood plasma). The albumin concentrations in the CSF collected from the third brain ventricle and blood plasma were calculated on the basis of a linear dilution curve of sheep serum albumin detected by the Western blot method (Supplementary Figure S7).

#### 4.2.4. Relative Gene Expression Assays

Total RNA from the ChP and ChP epithelial cells was isolated using NucleoSpin RNAII Kit (MARCHEREY-NAGEL, Düren, Germany) according to the manufacturer’s protocol. The step for genomic DNA digestion was included in the isolation procedure. The purity and concentration of the isolated RNA was quantified spectrophotometrically using a NanoDrop 1000 instrument (Thermo Fisher Scientific, Waltham, MA, USA). The RNA integrity was confirmed by electrophoresis using 1.2% agarose gel stained with GelRed^TM^ Nucleic Acid Gel Stain (Biotium, Fremont, CA USA). To synthetize cDNA, the Maxima First Strand cDNA Synthesis Kit for RT-qPCR (Thermo Fisher Scientific, Waltham, MA, USA) and 1 µg of total RNA were used. RT-PCR was performed using a DyNAmoSYBR Green qPCR Kit with ROX (Thermo Fisher Scientific, Waltham, MA, USA) with a Viia7 instrument (Applied Biosystems by Life Technologies, Waltham, MA, USA). Specific primer pairs for the different genes were used according to the literature [29,42,46,47,48,49,50,51,52], or were designed using Primer-BLAST (National Center for Biotechnology Information, Bethesda, MD, USA) and were synthesized by Genomed (Warsaw, Poland). The analyses of *GUSB* expression were conducted with the use of originally designed primer pairs for which sequences varied depending on the species: Frd 5′-GAGCGAGTACGGAGCAGATG-3′, Rev 5′-ATCATCCGAACTGGTGACTGG-3′, sheep, Frd 5′-GAGCAAGACAGTGGGCTGG-3′, Rev 5′-CCATTCGCCACGACTTTGTT-3′ human and Frd 5′-TGGCTGGGTGTGGTATGAAC-3′, Rev 5′-ATCCCATTCACCCACACAACT-3′ rats. All the sequences of the oligonucleotides used in the presented studies are detailed in Supplementary Table S3. For easier reception of the work, the nomenclature of genes was unified, and regardless of the species, all gene symbols were written in capital letters. For real-time PCR analysis, the following protocol was used: 95 °C for 10 min for the hot start modified Tbr DNA polymerase, followed by 40 cycles of 95 °C for 15 s (denaturation), primer annealing at 60 °C (human and sheep genes) or 55 °C (rat genes) for 30 s and 72 °C for 30 s (extension). The last cycle was performed to evaluate the specific amplification using the final melting curve until continuous fluorescence measurements. The reference genes and their combinations (geometric mean) were selected based on our previous experiences, and for the expression analysis glyceraldehyde-3-phosphate dehydrogenase (*GAPDH*), β-actin (*ACTB*) and histone deacetylase 1 (*HDAC1*) were used for sheep samples, *ACTB*, 3-monooxygenase/tryptophan 5-monooxygenase activation protein zeta (*YWHAZ)* and peptidylprolyl isomerase A (*PPIA)* were used for the rat ChP epithelial cells, and *GAPDH* was used for the human HIBCPP cells. The results were analyzed using Real-Time PCR Miner (available online: http://ewindup.info/miner/ (accessed on 10 May 2021)) based on the algorithm developed by Zhao and Fernald [53].

#### 4.2.5. Analysis of Q and Its Metabolites with HPLC-MS/MS

The quercetin metabolite (quercetin and isorhamnetin aglycones and their conjugate forms) analysis was performed on an LC-200 Eksigent HPLC system combined with an MS/MS QTRAP 5500 (AB SCIEX, Canada) working in the ESI mode according to the modified method of Wiczkowski et al. [16]. Briefly, after thawing, samples were extracted with a 2.5 times larger volume of methanol/formic acid (99/1; *v*/*v*) solution. The obtained mixture was subjected to 30 s vortexing and 30 s sonication followed by centrifugation at 13,200× *g* for 20 min. Extraction was performed in triplicate, each time collecting the supernatants which, after combining, were evaporated until dry in a nitrogen stream. The residue was dissolved in 50 μL of 50 % (*v*/*v*) methanol in water with formic acid (99.9/0.1; *v*/*v*), centrifuged at 13,200× *g* for 20 min, and injected into the HPLC-MS/MS system. The five microliters of samples were loaded on the HALO C_18_ column (0.5 mm × 50 mm × 2.7 µm, Eksigent, Toronto, ON, Canada) with the mobile phase flow set at 20 µL/min in a gradient composed of water/formic acid (99.9:0.1, phase A) and acetonitrile/formic acid (99.9:0.1, phase B) as follows: 2–2–95–95–2–2% of phase B at 0 –0.1–2–2.5–2.7–3.0 min, respectively. Optimal identification of quercetin metabolites was achieved under the following conditions: curtain gas, 25 L/min; collision gas, 9 L/min; ion spray voltage, −4500 V; temperature, 350 °C, one ion source gas, 30 L/min; two ion source gas, 35 L/min; de-clustering potential, −100 to 125 V; entrance potential, −10 V; collision energy, −26 to 40 eV, and collision cell exit potential: −12.5 to 25 V. Qualitative and quantitative analyses of quercetin compounds were performed by comparing their retention times and the presence of respective parent and daughter ion pairs (multiple reaction monitoring method, MRM) with appropriate external standards and literature data: 301–151 *m*/*z* and 301–179 *m*/*z* for quercetin, 315–300 *m*/*z* for isorhamnetin, 477–301 *m*/*z* for quercetin glucuronide, 491–315 *m*/*z* isorhamnetin glucuronide, 557–301 *m*/*z* for quercetin sulpho-glucuronide, 571–315 *m*/*z* for isorhamnetin sulpho-glucuronide, 381–301 *m*/*z* for quercetin sulphate, and 395–315 *m*/*z* for isorhamnetin sulphate [16]. The calibration curve (range of 1–100 nM) was linear with a correlation coefficient of 0.97–0.98. The sensitivities of the assay for Q3GA, Q, iR and iRGA are listed in Supplementary Table S4.

### 4.3. Calculations and Statistical Analysis

All statistical analyses were performed with Graph Pad PRISM 8 (GraphPad Software, San Diego, CA, USA). Before statistical analyses, all data were verified in terms of passing the normality test (Shapiro–Wilk test), and those data which failed were subjected to logarithmic transformation. All data are presented as the mean ± standard error of the mean (SEM).

The results of real-time PCR are presented in a normalized form as the relative expression of the tested genes compared to the geometric mean expression of the reference genes. The normalization consisted of bringing the average value obtained for one of the compared groups to a value close to 1 by dividing all partial results by the median value for this group. Relative gene expression (*GUSB*, *CCL2, CXCL1* and *IL8*) and β-Gluc enzyme activity in the ChP and in HIBCPP cells were analyzed by two-way ANOVA followed by post hoc Tukey’s test. Relative gene expression (*GUSB*, *IL6* and *CXCL1*) and β-Gluc activity in rat ChP epithelial cells were analyzed using the non-parametric Mann–Whitney U test comparing two independent groups. Analyses of the LPS and Q3GA toxicity tests were performed on the absorbance values and the results were expressed as percentages. Percentage data were multiplied by 0.1 and then arcsin-transformed according to the equation (Y = deg (arcsin (sqrt (Y/100)))). The transformed data were subjected to one-way ANOVA. A two-way ANOVA for repeated measures followed by post hoc Tukey’s test was used to analyze body temperature and cortisol concentrations, and β-Gluc activity in blood and CSF. The Q3GA concentrations in HIBCPP cells were analyzed by two-way ANOVA followed by post hoc Tukey’s test. The relationship between Q3GA dose and Q3GA concentration in HIBCPP cells was analyzed using the Spearman’s correlation coefficient. A two-way ANOVA for repeated measures followed by post hoc Dunnett’s and Sidak’s test was used to analyze the concentration of Q and its metabolites in the blood plasma and CSF. The proportions of Q and its metabolites in blood plasma and CSF are presented as percentages. Total Q3GA, Q, iR and iRGA, measured by the area under the concentration of Q3GA, Q, iR and iRGA over time (AUC) in blood plasma and CSF, was analyzed using the non-parametric Wilcoxon paired test. Statistical significance was assumed at *p* < 0.05.

## Data Availability

Data are available from the corresponding author upon reasonable request.

## Supplementary Materials

Supplementary materials can be found at https://www.mdpi.com/article/10.3390/ijms22137122/s1.
