# Supplementary material for: Effect of Lipopolysaccharide-Induced Inflammatory Challenge on β-Glucuronidase Activity and the Concentration of Quercetin and Its Metabolites in the Choroid Plexus, Blood Plasma and Cerebrospinal Fluid
Source: Int J Mol Sci. 2021 Jul 1;22(13):7122. doi: 10.3390/ijms22137122 (PMC8268849; doi:10.3390/ijms22137122)
Supplement: Supplementary file 1 [file ijms-22-07122-s001.zip › ijms-1249983-supplementary.pdf]

Effect of Lipopolysaccharide-induced Inflammatory Challenge on β-glucuronidase Activity and Quercetin and its Metabolites Concentration in the Choroid Plexus, Blood Plasma and Cerebrospinal Fluid

Małgorzata Domżalska, Wiesław Wiczkowski, Aleksandra Szczepkowska, Sylwia Chojnowska, Tomasz Misztal, Fruzsina R. Walter, Maria A. Deli, Hiroshi Ishikawa, Horst Schroten, Christian Schwerk and Janina Skipor

SUPPLEMENTARY MATERIALS

Supplementary Figure S1: Body temperature values and cortisol concentration in blood plasma throughout experiment 1.

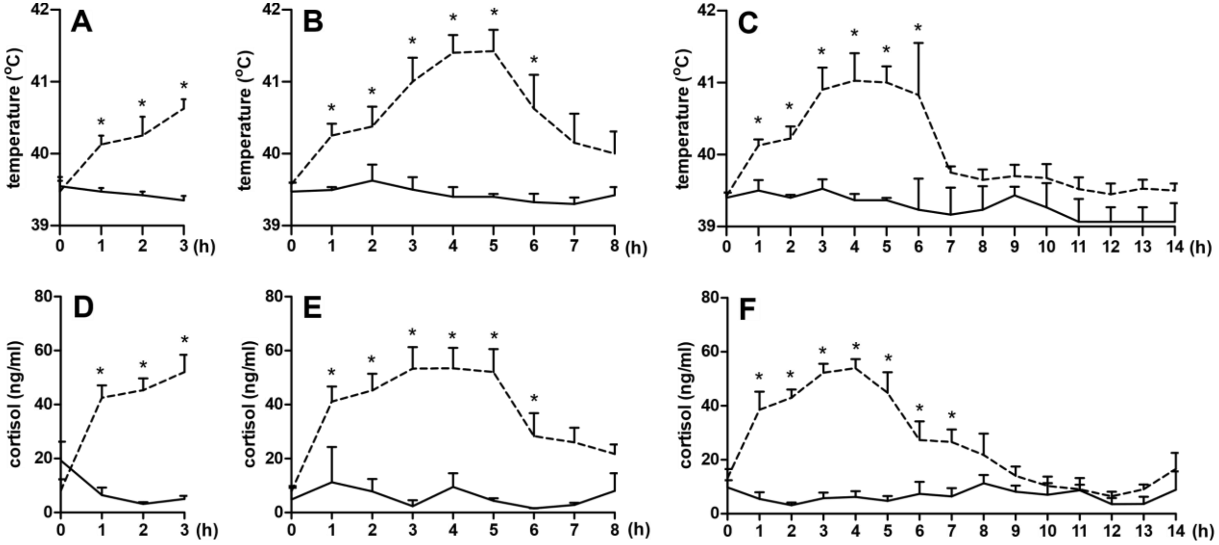

Figure S1. Mean (±SEM) body temperature and plasma cortisol concentration in saline-treated (solid line) and lipopolysaccharide (LPS)-treated (dotted line) ewes euthanized 3 h (A, D), 8 h (B, E) and 14 h (C, F) after saline/LPS administration in experiment 1. * indicate significant differences at $p < 0.05$ (two-way ANOVA—Tukey's post hoc test).

Supplementary Figure S2: *CCL2* and *CXCL1* gene expression in the ovine ChP.

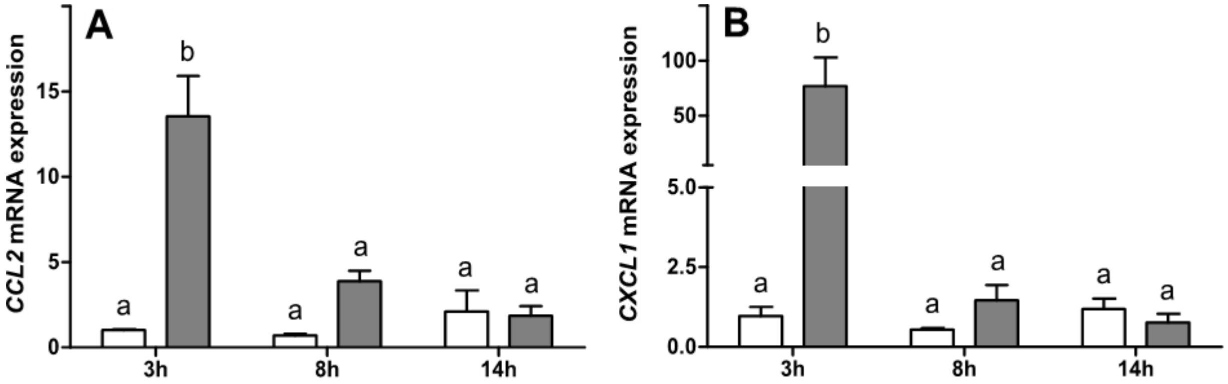

Figure S2. Mean (±SEM) relative mRNA expression of *CCL2* (A) and *CXCL1* (B) in the ovine choroid plexus, 3, 8, and 14 h after saline (white bars) or lipopolysaccharide (LPS, grey bars) administration. Different lower case letters indicate significant differences at $p < 0.05$ (two-way ANOVA—Tukey's post hoc test).

Supplementary Figure S3: Body temperature values and cortisol concentration in blood plasma throughout experiment 2.

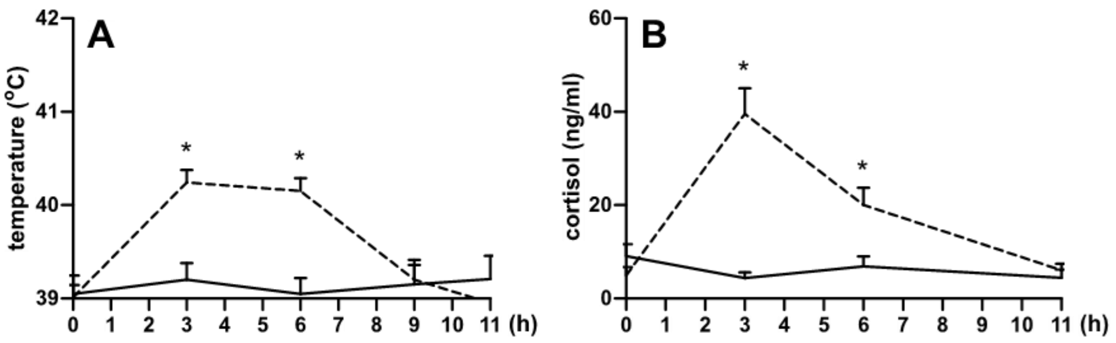

Figure S3. Mean (±SEM) body temperature (A) and plasma cortisol concentration (B) in ewes treated with saline (solid line) or lipopolysaccharide (LPS, dotted line) in experiment 5. *- indicate significant differences at $p < 0.05$ (two-way ANOVA—Tukey's post hoc test).

Supplementary Figure S4: CSF/serum albumin ratio (Q-Alb) throughout experiment 2.

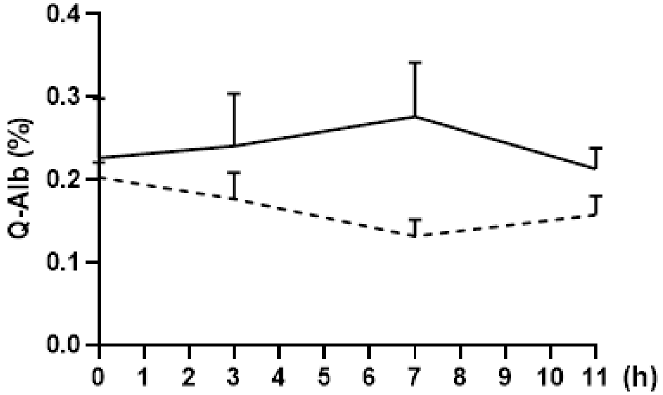

Figure S4. Mean (±SEM) cerebrospinal fluid/serum albumin ratio (Q-Alb) in ewes treated with saline (solid line) or lipopolysaccharide (LPS, dotted line) in experiment 5 (two-way ANOVA—Tukey’s post hoc test).

Supplementary Table S1: The % proportion of quercetin derivatives detected in sheep blood plasma over time after an intravenous administration of Q3GA at a dose of 6 mg/kg bw in saline- (C) and LPS-treated group.

	time (min)																				total (%)	
	0		30		60		120		180		240		300		360		420		480			
	C	LPS	C	LPS	C	LPS	C	LPS	C	LPS	C	LPS	C	LPS	C	LPS	C	LPS	C	LPS	C	LPS
**Q3GA**	1,54	0,12	65,88	77,58	3,32	0,20	3,57	0,80	2,94	0,34	1,41	0,14	1,50	0,18	1,72	0,21	1,47	0,10	1,00	0,14	84,36	79,82
**Q**	2,63	0,21	0,53	2,30	1,16	1,99	2,44	3,06	3,22	3,14	2,16	0,25	1,25	0,10	0,19	3,16	0,47	2,08	0,09	3,50	14,15	19,79
**iR**	0,06	0,06	0,19	0,15	0,16	0,01	0,09	0,02	0,16	0,02	0,03	0,01	0,31	0,02	0,09	0,02	0,19	0,00	0,12	0,05	1,40	0,37
**iRGA**	0,01	0,00	0,01	0,00	0,02	0,00	0,01	0,00	0,01	0,00	0,01	0,01	0,01	0,00	0,01	0,00	0,01	0,00	0,01	0,00	0,10	0,03
**total (%)**	4,25	0,39	66,61	80,03	4,65	2,20	6,11	3,89	6,33	3,51	3,60	0,40	3,07	0,31	2,01	3,39	2,14	2,19	1,22	3,69	100,00	100,00

Supplementary Table S2: The % proportion of quercetin derivatives detected in sheep cerebrospinal fluid over time after an intravenous administration of Q3GA at a dose of 6 mg/kg bw in saline- (C) and LPS-treated group.

	time (min)																		total (%)	
	0		60		120		180		240		300		360		420		480			
	C	LPS	C	LPS	C	LPS	C	LPS	C	LPS	C	LPS	C	LPS	C	LPS	C	LPS	C	LPS
**Q3GA**	4,35	6,07	6,81	62,29	6,14	1,48	2,73	0,87	11,15	1,02	7,77	0,26	10,32	1,94	3,16	0,77	2,22	0,10	54,65	74,79
**Q**	2,94	3,32	3,99	2,50	4,84	1,58	3,70	1,68	4,34	2,55	3,73	0,97	13,03	3,37	3,34	2,24	3,91	4,03	43,83	22,24
**iR**	0,09	0,36	0,03	0,41	0,05	0,20	0,08	0,10	0,05	0,15	0,08	0,15	0,05	0,20	0,03	0,26	0,02	0,10	0,46	1,94
**iRGA**	0,09	0,10	0,14	0,41	0,21	0,05	0,02	0,01	0,09	0,05	0,20	0,15	0,12	0,26	0,11	0,01	0,09	0,01	1,06	1,04
**total (%)**	7,48	9,84	10,97	65,61	11,24	3,32	6,52	2,66	15,63	3,77	11,77	1,53	23,52	5,76	6,63	3,27	6,24	4,24	100,00	100,00

Supplementary Figure S5: Validation of rat ChP epithelial cells isolation and inflammatory response to LPS stimulation

.

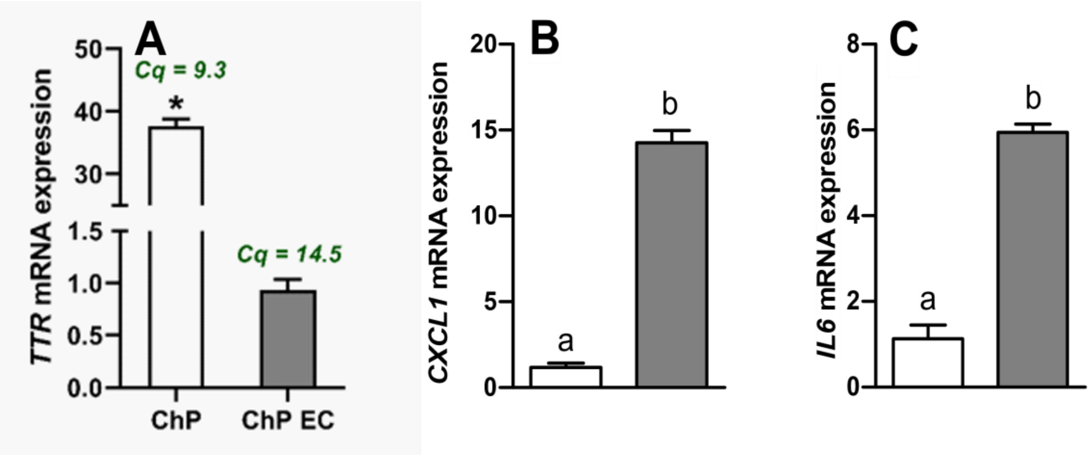

Figure S5. (A) Mean (±SEM) *TTR* mRNA expression in choroid plexus (ChP, white bar) and choroid plexus epithelial cells (ChP EC, gray bar) of male rats. * indicate significant differences at $p < 0.05$ (Mann-Whitney U test); Cq – cycle quantification value, (n=3). **(B, C)** Mean (± SEM) relative mRNA expression of *CXCL1* (B) and *IL6* (C) in rat ChP epithelial cells after 3 h incubation with saline (white bars) or lipopolysaccharide (LPS, 5 μg/ml, grey bars), (n=3). Different lower case letters indicate significant differences at $p < 0.05$ (Mann-Whitney U test).

Supplementary Figure S6: Response of HIBCPP cell line on LPS treatment.

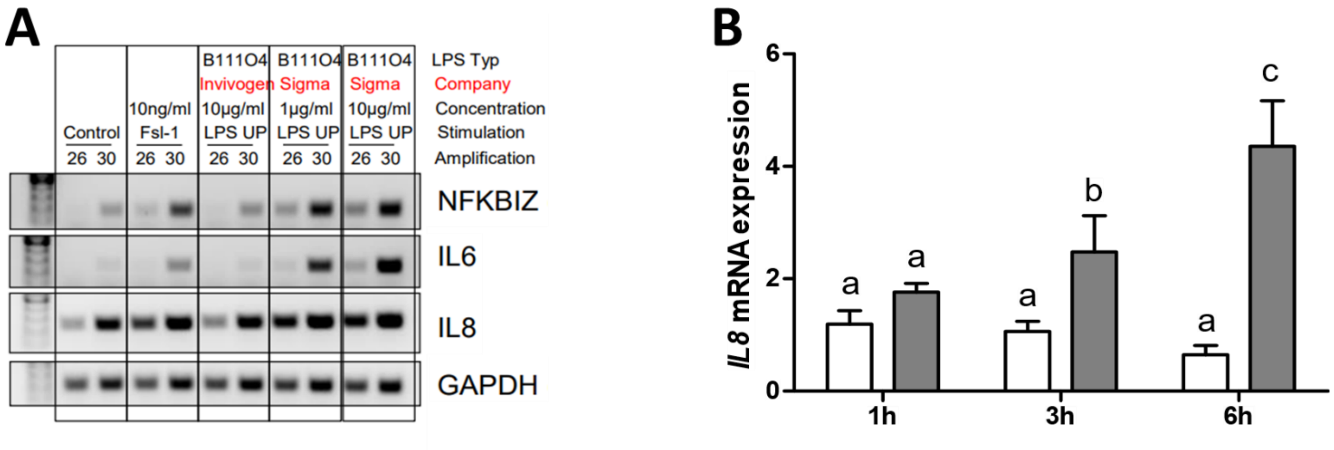

Figure S6. (**A**) regular PCR results showing mRNA expression of *NFKBIZ* (line 1), *IL6* (line 2), *IL8* (line 3), and reference gene *GAPDH* (line 4) in saline, Fsl-1, and lipopolysaccharide (LPS, No: tlrl-eblps Invivogen, Toulouse France; No: L3024-5MG Sigma Aldrich, St. Louis, MO, USA) treated HIBCPP cells.
(**B**) Mean (±SEM) relative mRNA expression of *IL8* in the HIBCPP cell line after 1, 3, and 6 h incubation with saline (white bars) or lipopolysaccharide (LPS, 5 µg/ml, grey bars), (n=3). Different lower case letters indicate significant differences at $p < 0.05$ (two-way ANOVA—Tukey's post hoc test).

Supplementary Table S3: Sequences of primer pairs used for RT-PCR.

Gene	(Forward/ Reverse) Sequence 5'→3'		Amplicon size (bp)	References/ Sources
**Human**				
GUSB	F: GAG CAA GAC AGT GGG CTG G	R: CCA TTC GCC ACG ACT TTG TT	107	NM_000181.4
IL8	F: CAA GAG CCA GGA AGA AAC CA	R: GTC CAC TCT CAA TCA CTC TCA G	225	Borkowski et al., 2014
IL6	F: AAC CTG AAC CTT CCA AAG ATG G	R: TCT GGC TTG TTC CTC ACT ACT	159	Borkowski et al., 2014
NFKBIZ	F: CAG TTC AAG TTA GCT GGC TGA	R: TCT GTG GAG AAT ACT GGT ACA GG	177	Borkowski et al., 2014
GAPDH*	F: TGT TGC CAT CAA TGA CCC CTT	R: CTC CAC GAC GTA CTC AGC G	202	Borkowski et al., 2014
**Rat**				
GUSB	F: TGG CTG GGT GTG GTA TGA AC	R: ATC CCA TTC ACC CAC ACA ACT	123	NM_017015.2
IL6	F: TCC TAC CCC AAC TTC CAA TGC TC	R: TTG GAT GGT CTT GGT CCT TAG CC	79	Peinnequin et al., 2004
CXCL1	F: GGC AGG GAT TCA CTT CAA GA	R: GCC ATC GGT GCA ATC TAT CT	204	Malik et al., 2010
TTR	F: ATG GTC AAA GTC CTG GAT GC	R: GCC AAG AGC CTT CCA GTA TG	213	Sheng et al., 2016
ACTB*	F: TGT CAC CAA CTG GGA CGA TA	R: GGG GTG TTG AAG GTC TCA AA	165	Bonefeld et al., 2008
YWHAZ*	F: TTC GCA GCC AGA AAG CAA AG	R: TTG TCA TCA CCA GCA GCA AC	87	Takizawa et al., 2016
PPIA*	F: TAT CTG CAC TGC CAA GAC TGA GTG	R: CTT CTT GCT GGT CTT GCC ATT CC	126	Langnaese et al., 2008
**Sheep**				
GUSB	F: GAG CGA GTA CGG AGC AGA TG	R: ATC ATC CGA ACT GGT GAC TGG	191	NM_001083436.1
CCL2	F: CTC GCT CAG CCA GAT GCA AT	R: AGG TTG GGG TCT GCA CAA AA	173	Szczepkowska et al., 2020
CXCL1	F: ATG CAG AGC GTG AAG GTG AC	R: GTT GGA GCT GGC CTG GTT T	153	NM_001009358.1
GAPDH*	F: TGA CCC CTT CAT TGA CCT TC	R: GAT CTC GCT CCT GGA AGA TG	143	Kowalewska et al., 2017
ACTB*	F:GCC AAC CGT GAG AAG ATG AC	R: TCC ATC ACG ATG CCA GTG	122	Kowalewska et al., 2017
HDAC1*	F: CTG GGG ACC TAC GGG ATA TT	R: GAC ATG ACC GGC TTG AAA AT	115	Kowalewska et al., 2017

GUSB – β-glucuronidase; *IL8* – interleukin 8; *IL6* – interleukin 6; *CXCL1* – C-X-C motif chemokine ligand 1; *CCL2* – C-C motif chemokine ligand 2; *TTR* – transthyretin; *reference genes: *GAPDH* – glyceraldehyde-3-phosphate dehydrogenase; *ACTB* – beta-actin; *YWHAZ* – tyrosine 3-monooxygenase/tryptophan 5-monooxygenase activation protein zeta; *PPIA* – peptidylprolyl isomerase A; *HDAC1* – histone deacetylase1. Exon/exon span: human *GUSB* forward primer – F:754/755; rat *GUSB* reverse primer – R:432/433; sheep *GUSB* reverse primer – R:2038/2038; sheep *CXCL1* reverse primer – R:322/323.

Supplementary Figure S7: Linear dilution curve of sheep serum albumin and cerebrospinal fluid and blood plasma albumin levels detected by western blot method

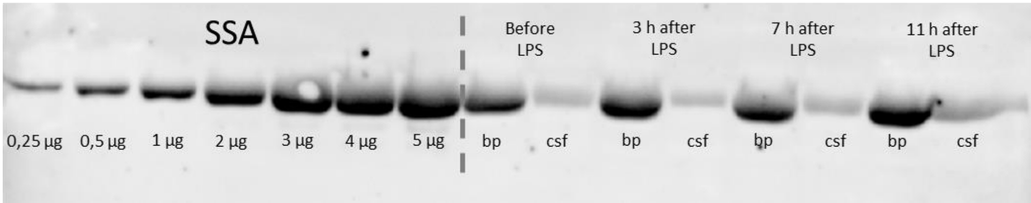

Figure S7. Determination of albumin levels by western blot analysis in sheep blood plasma (bp) and cerebrospinal fluid (csf) samples collected before and 3, 7 and 11 h after LPS administration. The densities of the bands were based on sheep serum albumin (SSA) dilution curve.

Supplementary Table S4: Sensitivity of the HPLC-MS/MS assay for Q3GA, Q, iR and iRGA for blood plasma, cerebrospinal fluid (CSF) and tissues/ cells.

	**Q3GA**	**Q**	**iR**	**iRGA**
**blood plasma**	0,00192 nmol/L	0,00046 nmol/L	0,00019 nmol/L	0,00014 nmol/L
**CSF**	0,00226 nmol/L	0,00132 nmol/L	0,00054 nmol/L	ND*
**tissues/cells**	0,00155 nmol/g	0,00053 nmol/g	0,00035 nmol/g	ND*

ND* - no data

References for Supplementary Materials:

1. Borkowski, J.; Li, L.; Steinmann, U.; Quednau, N.; Stump-Guthier, C.; Weiss, C.; Findeisen, P.; Gretz, N.; Ishikawa, H.; Tenenbaum, T.; Schroten, H.; Schwerk, C. Neisseria meningitidis elicits a pro-inflammatory response involving IκBζ in a human blood-cerebrospinal fluid barrier model. *J Neuroinflammation*. **2014**, 11:163.
2. Peinnequin, A.; Mouret, C.; Birot, O.; Alonso, A.; Mathieu, J.; Clarençon, D.; Agay, D.; Chancerelle, Y.; Multon, E. Rat pro-inflammatory cytokine and cytokine related mRNA quantification by real-time polymerase chain reaction using SYBR green. *BMC Immunol*. **2004**, 5:3.
3. Malik, I.A.; Moriconi, F.; Sheikh, N.; Naz, N.; Khan, S.; Dudas, J.; Mansuroglu, T.; Hess, C.F.; Rave-Fränk, M.; Christiansen, H.; Ramadori, G. Single-dose gamma-irradiation induces up-regulation of chemokine gene expression and recruitment of granulocytes into the portal area but not into other regions of rat hepatic tissue. *Am J Pathol*. **2010**, 176(4):1801–1815.
4. Zheng, F.; Kim, Y.J.; Moran, T.H., Li, H.; Bi, S. Central transthyretin acts to decrease food intake and body weight. *Sci Rep*. **2016**, *6*, 24238.
5. Bonefeld, B.E.; Elfving, B.; Wegener, G. Reference genes for normalization: a study of rat brain tissue. *Synapse*. **2008**, 62(4):302–309.
6. Takizawa, N.; Tanaka, S.; Oe, S.; Koike, T.; Matsuda, T.; Yamada, H. Hypothalamo-hypophysial system in rats with autotransplantation of the adrenal cortex. *Mol Med Rep*. **2017**, 15(5):3215–3221.
7. Langnaese, K.; John, R.; Schweizer, H.; Ebmeyer, U.; Keilhoff, G. Selection of reference genes for quantitative real-time PCR in a rat asphyxial cardiac arrest model. *BMC Mol Biol*. **2008**, 9:53.
8. Szczepkowska, A.; Kowalewska, M.; Krawczyńska, A.; Herman, A.P.; Skipor, J. Photoperiod affects leptin action on the choroid plexus in ewes challenged with lipopolysaccharide – study on the mRNA level. *IJMS*. **2020**, 21(20):1–16.
9. Kowalewska, M.; Herman, A.P.; Szczepkowska, A.; Skipor, J. The effect of melatonin from slow-release implants on basic and TLR-4-mediated gene expression of inflammatory cytokines and their receptors in the choroid plexus in ewes. *Res Vet Sci*. **2017**, 113:50–55.
